# Attitudes toward immigration in Europe: Cross-regional differences

**DOI:** 10.12688/openreseurope.15691.1

**Published:** 2023-04-28

**Authors:** Lenka Dražanová, Jérôme Gonnot

**Affiliations:** 1 Migration Policy Centre, European University Institute, Florence, I-50014, Italy; 2 Centre d'Etudes Prospectives et d'Informations Internationales (CEPII), Paris, 75334, France

**Keywords:** attitudes to immigration, migration flows, public opinion, regions

## Abstract

**Background: **This article investigates how European public opinion has responded to short-term variations in regional immigration over the past decade (2010-2019).

**Methods: **Combining data from the European Social Survey and the European Union Labour Force Survey and using multilevel modelling, we test how natives’ opinions over migration policy and the contribution of immigrants to society have changed with the net rate of international migrants in 183 EU regions from 21 countries.

**Results: **We find that while European natives living in regions with a higher share of foreign-born populations are generally less anti-immigrant, a short-term increase in the number of immigrants within a given region is associated with more negative attitudes.

**Conclusion: **Our findings demonstrate the importance of temporal dynamics for attitudes to immigration. They also point to the relevance of regional variations in attitudes beside cross-country differences.

## 1. Introduction

Attitudes to immigration are becoming part of a new political cleavage in many countries ( Kriesi *et al.,* 2012). While a growing share of foreign-born residents is viewed positively by those stressing the benefits of immigration, others regard these demographic changes with suspicion. Especially in the aftermath of the so-called “migration crisis”, governments of Western as well as Central and Eastern European countries, though historically on the sending side of immigration, have faced public resentment against immigrants among their domestic population.

Against this backdrop, opposition to immigration has gained a lot of attention from social scientists. While the majority of studies have focused on individual drivers of attitudes to immigration (see Dražanová *et al.*, 2022 for a meta-analysis), the scientific literature has shown that contextual drivers, and in particular, the real or perceived size of immigration can have a significant influence on public opinion (see for instance Alesina *et al.*, 2018). At the same time, several recent studies have documented the role played by immigrants’ characteristics as potential drivers of attitudes towards migration in Europe ( Bridges & Mateut, 2014; Hale Williams & Chasapopoulos, 2019; Markaki & Longhi, 2013; Weber, 2015). This work contributes to this literature by exploring the link between the temporary changes in migration flows in European regions on individuals’ attitudes towards immigration and deepening our understanding of the macro-level drivers of attitudes to migration in European countries.

Previous empirical research has examined the impact of regional factors on attitudes towards immigrants in Europe, and in particular how the size of immigration and the characteristics of immigrants predict attitudes to immigration. In this regard, our paper is similar to Markaki and Longhi (2013) and Hale Williams and Chasapopoulos (2019). However, we differentiate ourselves from these studies in several ways. While these works focus on the effect of between-region variations in the share of foreign-born immigrants, we primarily consider how short-term, temporal within-region variations predict attitudes to immigration. Traditionally, the share of the foreign-born population residing in a territory is usually the product of long-term changes and migration history, whose effects can be hard to disentangle from other macro-level, contextual drivers of attitudes to immigration such as economic conditions, cultural and religious beliefs, as well as national or regional policies. In this regard, we believe the predictive power of immigration on public opinion is better identified by focusing on migration pressure, or how natives’ attitudes towards immigration change with the recent arrival of foreign-born immigrants. In particular, we focus our attention on within-region, short-term temporal variations in the regional share of foreign-born immigrants.

A few studies have examined the impact of migration flows on natives’ attitudes towards preferences for redistribution (see for instance Murard, 2017) or voting behaviour ( Moriconi *et al.*, 2019). Others have studied more specifically their effect on support for far-right parties ( Brunner & Kuhn, 2018; Dustmann *et al.*, 2019; Halla *et al.,* 2017; Moriconi *et al.*, 2022). Only a handful of papers, however, investigate the relationship between natives’ exposure to short-term variations in the presence of foreign-born individuals and their attitudes towards immigrants. Among them, Karreth *et al.* (2015) find that increasing diversity is associated with negative attitudes toward immigrants among natives on the political right, while Newman and Velez (2014) document how rapidly growing immigration can lead to increased hostility when immigrants are perceived as a threat by the native population. Our paper extends this line of research by looking at the predictive power of regional migration flows on attitudes towards immigration at the European level, which has not yet been studied. One exception is Murard (2017), who examines the impact of immigration on preferences for redistribution and attitudes towards migration policy, finding a positive correlation between the arrival of migrants and anti-immigration attitudes between 2002 and 2012. Unlike him, we focus our attention on the past decade (2010–2019), a period when European countries experienced major economic turbulences and rising immigration.

We ask the following research question: How do regional temporal variations in flows of foreign-born migrants predict changes in natives’ attitudes about migration policy and their assessment of migrants’ economic, cultural and overall contribution to society?

Our analysis combines individual-level information with regional-level data from various sources. To measure immigration attitudes, we use the European Social Survey (ESS) data from rounds 5 to 9 and build two indices about natives’ attitudes to immigration. Firstly, their policy preference regarding levels of immigration. Secondly, their assessment of the economic, cultural, and overall contribution of immigration to their country. The data cover 97,193 individual respondents surveyed between 2010 and 2019 in 183 regions across 21 European countries. Our measure of regional migrant flows captures short-term variations in the share of foreign-born individuals at the NUTS2 regional level, obtained from the European Labour Force Survey. We also build on the recent literature on the determinants of public attitudes to immigration and control for individual drivers as well as contextual, region-specific factors such as GDP, unemployment rate and population density.

Our goal is to explain the differences in individual attitudes to immigration through variations in the share of immigrants within European regions and across time. The complexity of our design requires an accurate specification of influential factors at each level of analysis. In the present research, the data has a four-level hierarchical structure with individuals (micro-level) nested in region-years, regions and countries (macro-level). When, as here, nested data across multiple levels of analysis are present, it is appropriate, both theoretically and statistically, to employ multilevel models. We apply four-level random effects multilevel models that allow the estimation of effects based on intra-regional differences over time and stable differences between regions ( Bell *et al.*, 2019; Fairbrother, 2014). Immigration in Europe occurs not only across countries but also across regions within countries. To maximize the variation in immigrant shares across regions at the highest possible level of granularity, we focus on NUTS2 regions whenever possible.

Our findings reveal a statistically significant and positive association between attitudes to immigration and immigrants’ historical presence in the European Union – as measured through the share of foreign-born population over the past decade. ^1^ In contrast, short-term increases in the share of foreign-born immigrants are correlated with more negative attitudes on both migration policy as well as natives’ assessment of immigrants’ contribution to the country.

Our paper makes a direct contribution to the studies looking at the relationship between immigrants’ presence and public opinion on immigration in Europe. Hatton (2016) finds that pro-immigration opinion is negatively related to the share of immigrants living in a country. At the regional level, several empirical papers examine the impact of immigrants’ presence on attitudes towards immigrants ( Bridges & Mateut, 2014; Green *et al.*, 2010; Markaki & Longhi, 2013; Rustenbach, 2010; Weber, 2015; and Hale Williams & Chasapopoulos, 2019). For instance, Weber (2015)’s results show a negative correlation between the national proportion of immigrants and perceived threat. Across European NUTS1 regions, both Markaki and Longhi (2013) and Hale Williams and Chasapopoulos (2019) find that regions with a higher percentage of immigrants born outside the EU have a higher probability that natives express negative attitudes to immigration. Among the few papers investigating local migration flows, Kawalerowicz (2021) finds that anti-immigrant attitudes in the UK are more likely to be expressed by natives who live in constituencies where there has been a large change in diversity between 2001 and 2011. On the same topic, Karreth *et al.* (2015) show that increasing and visible diversity in Austria, Germany, and Switzerland is associated with negative attitudes toward immigrants, but only among natives on the political right. Like us, Murard (2017) studies the effect of regional flows of international migrants on preferences regarding migration policy. He finds that where immigrants tend to compete with natives for jobs due to similar skills or occupations, natives prefer policies that support welfare and put restrictions on migration.

Finally, this work is related to a recent working paper by Di Iasio and Wahba (2021), which proposes a symmetric approach to ours and studies the causal impact of attitudes to immigration on migration flows. Their findings indicate a negative causal relationship between anti-immigration attitudes and migration inflows to the EU. If natives’ hostility acts as a deterrent for migrants, this reinforces concerns about the self-selection of immigrants to areas where natives’ have more positive views on immigration.

The next section briefly introduces the theoretical framework on which we build to explore the relationship between regional migration and public opinion. We then present the data and our empirical strategy in Section 3. Our findings are discussed in Section 4. We conclude and discuss some opportunities for further research in Section 5.

## 2. Theoretical background

This paper builds on the large body of literature on the determinants of attitudes to immigration. Natives’ fears over immigration are usually regarded as a mix of economic and cultural concerns.

The theory of economic competition posits that natives and immigrants are economic rivals. In the labour market, this implies that immigration is perceived by natives as a threat to wages and job security ( Citrin *et al.*, 1997; Facchini & Mayda, 2012; Scheve & Slaughter, 2001). Negative perceptions about immigrants also appear to be driven by the fear that foreigners represent a net fiscal burden ( Boeri, 2010; Dustmann & Preston, 2007), leading to restrictive preferences about redistribution and effectively lower public spending in some instances ( Razin *et al.*, 2002; Speciale, 2012). Several works have shown that the perceived economic threat from immigrants plays a substantial part in driving natives’ attitudes ( Facchini & Mayda, 2009; Hainmueller & Hiscox, 2010; Hanson *et al.*, 2007; Pardos-Prado & Xena, 2019; Scheve & Slaughter, 2001).

The cultural threat, or conflict, theory, postulates that natives perceive immigrants as a challenge to their ethnicity and values. It holds that observable differences lead to discrimination and often hostility between groups with a preference for their own ethnicity ( Gorodzeisky & Semyonov, 2016; Hainmueller & Hiscox, 2007). As a result, where immigrants are socio-ethnically different, their arrival may upset the demographic and social structure of society and elicit more negative responses (see for instance Hainmueller and Hangartner, 2013) or increased support for xenophobic, far-right parties (see for instance Barone *et al.*, 2016). Symmetrically, it is important to highlight how the context of immigration can also improve public opinion: According to the contact theory, a larger immigrant group can increase the incidence of contact between natives and newcomers at the local level, therefore reducing prejudice and the perception of threat in the long run. In this regard, the work of Coenders and Scheepers (2008) and Hopkins (2010) suggest that negative reactions to immigrants are most likely in response to competition from recent foreign arrivals, rather than existing ethnic diversity. Therefore, natives who have been recently exposed to immigrants, and experienced a rapid increase in the number of immigrants living around them are likely to be immune to prejudice-reducing contact with immigrants, while feelings of economic or/and ethnic competition are then more likely to emerge.

## 3. Methods

In this paper, we combine data from multiple sources to create a dataset that includes individual-level information on native individuals’ attitudes toward immigration and several regional variables.

At the individual level, the present analysis relies on biannual data from the European Social Survey (ESS). It contains 97,193 respondents from 21 European countries across 183 regions. Because our primary objective is to identify how public opinion reacts to short-term, within-region changes in attitudes to immigration, we only include in our analysis countries surveyed by the ESS at least twice over the time period under scrutiny (2010–2019). ^2^ Using the ESS allows us to disentangle attitudes to immigration across a number of European regions and within regions across time because people of the same region are observed at different time periods. Table 1 below shows the number of respondents for each region and each ESS round included in the sample.

**Table 1.** Number of observations per region and year.

Country	Region	2010	2012	2014	2016	2018	Total
Austria (AT)	AT1	863	0	615	0	916	2394
	AT2	435	0	332	0	518	1285
	AT3	724	0	637	0	791	2152
Belgium (BE)	BE10	74	0	89	0	66	229
	BE21	281	0	224	0	255	760
	BE22	147	0	133	0	145	425
	BE23	207	0	195	0	195	597
	BE24	159	0	140	0	140	439
	BE25	194	0	217	0	210	621
	BE31	49	0	57	0	67	173
	BE32	154	0	199	0	178	531
	BE33	137	0	168	0	131	436
	BE34	42	0	50	0	41	133
	BE35	72	0	70	0	61	203
Bulgaria (BG)	BG31	366	0	0	0	285	651
	BG32	334	0	0	0	271	605
	BG33	322	0	0	0	290	612
	BG34	403	0	0	0	305	708
	BG41	537	0	0	0	544	1081
	BG42	450	0	0	0	487	937
Czech Republic (CZ)	CZ01	277	0	254	0	319	850
	CZ02	230	0	249	0	276	755
	CZ03	262	0	250	0	251	763
	CZ04	235	0	256	0	266	757
	CZ05	345	0	347	0	328	1020
	CZ06	388	0	281	0	375	1044
	CZ07	310	0	191	0	256	757
	CZ08	293	0	275	0	267	835
Germany (DE)	DE1	255	0	295	0	228	778
	DE2	371	0	355	0	356	1082
	DE3	89	0	140	0	73	302
	DE4	224	0	179	0	62	465
	DE5	22	0	22	0	6	50
	DE6	21	0	41	0	42	104
	DE7	162	0	162	0	124	448
	DE8	116	0	120	0	51	287
	DE9	168	0	222	0	244	634
	DEA	487	0	427	0	414	1328
	DEB	117	0	112	0	97	326
	DEC	19	0	30	0	23	72
	DED	248	0	261	0	133	642
	DEE	177	0	146	0	66	389
	DEF	77	0	78	0	79	234
	DEG	192	0	156	0	58	406
Denmark (DK)	DK01	364	0	354	0	172	890
	DK02	200	0	201	0	351	752
	DK03	332	0	341	0	349	1022
	DK04	382	0	346	0	393	1121
	DK05	197	0	142	0	204	543
Spain (ES)	ES11	97	0	133	0	125	355
	ES12	42	0	51	0	37	130
	ES13	23	0	30	0	23	76
	ES21	75	0	89	0	74	238
	ES22	27	0	24	0	15	66
	ES23	6	0	16	0	9	31
	ES24	55	0	58	0	49	162
	ES30	265	0	211	0	169	645
	ES41	110	0	105	0	86	301
	ES42	77	0	85	0	88	250
	ES43	52	0	52	0	45	149
	ES51	208	0	222	0	180	610
	ES52	159	0	181	0	142	482
	ES53	23	0	35	0	21	79
	ES61	374	0	336	0	295	1005
	ES62	42	0	50	0	35	127
	ES63	0	0	4	0	4	8
	ES64	2	0	5	0	3	10
	ES70	56	0	69	0	60	185
Finland (FI)	FI19	484	0	544	0	437	1465
	FI1B	454	0	504	0	446	1404
	FI1C	416	0	424	0	363	1203
	FI1D	459	0	502	0	421	1382
	FI20	0	0	13	0	4	17
France (FR)	FR10	209	0	216	0	213	638
	FRB0	55	0	75	0	75	205
	FRC1	37	0	48	0	55	140
	FRC2	42	0	19	0	38	99
	FRD1	46	0	51	0	49	146
	FRD2	49	0	47	0	50	146
	FRE1	114	0	84	0	122	320
	FRE2	34	0	78	0	70	182
	FRF1	41	0	58	0	54	153
	FRF2	45	0	17	0	31	93
	FRF3	88	0	42	0	56	186
	FRG0	103	0	104	0	118	325
	FRH0	91	0	130	0	122	343
	FRI1	90	0	111	0	110	311
	FRI2	28	0	45	0	24	97
	FRI3	84	0	40	0	51	175
	FRJ1	54	0	77	0	71	202
	FRJ2	68	0	125	0	85	278
	FRK1	35	0	44	0	44	123
	FRK2	169	0	126	0	185	480
	FRL0	92	0	157	0	146	395
Croatia (HR)	HR03	493	0	0	0	586	1079
	HR04	989	0	0	0	1042	2031
Hungary (HU)	HU11	0	0	294	0	203	497
	HU12	0	0	225	0	227	452
	HU21	181	0	176	0	210	567
	HU22	160	0	151	0	155	466
	HU23	170	0	154	0	103	427
	HU31	183	0	207	0	217	607
	HU32	255	0	259	0	296	810
	HU33	183	0	205	0	231	619
Ireland (IE)	IE04	0	0	533	0	424	957
	IE05	0	0	725	0	636	1361
	IE06	0	0	819	0	763	1582
Italy (IT)	ITC1	0	62	0	206	0	268
	ITC2	0	0	0	32	0	32
	ITC3	0	24	0	38	0	62
	ITC4	0	79	0	335	0	414
	ITF1	0	35	0	26	0	61
	ITF3	0	80	0	217	0	297
	ITF4	0	22	0	230	0	252
	ITF5	0	43	0	50	0	93
	ITF6	0	70	0	59	0	129
	ITG1	0	121	0	185	0	306
	ITG2	0	35	0	66	0	101
	ITH1	0	15	0	19	0	34
	ITH2	0	10	0	10	0	20
	ITH3	0	60	0	204	0	264
	ITH4	0	5	0	69	0	74
	ITH5	0	55	0	257	0	312
	ITI1	0	68	0	97	0	165
	ITI2	0	13	0	37	0	50
	ITI3	0	19	0	87	0	106
	ITI4	0	74	0	171	0	245
Lithuania (LT)	LT01	0	0	527	0	409	936
	LT02	0	0	1648	0	1370	3018
Norway (NO)	NO01	273	0	282	0	301	856
	NO02	102	0	93	0	79	274
	NO03	272	0	256	0	219	747
	NO04	222	0	155	0	177	554
	NO05	252	0	227	0	238	717
	NO06	143	0	130	0	109	382
	NO07	132	0	124	0	134	390
Poland (PL)	PL12	227	0	225	0	174	626
	PL21	141	0	162	0	152	455
	PL22	204	0	175	0	220	599
	PL41	146	0	139	0	107	392
	PL42	79	0	64	0	51	194
	PL43	39	0	43	0	34	116
	PL51	109	0	85	0	102	296
	PL52	50	0	31	0	33	114
	PL61	101	0	96	0	77	274
	PL62	64	0	50	0	62	176
	PL63	115	0	80	0	78	273
	PL71	125	0	129	0	118	372
	PL72	69	0	62	0	48	179
	PL81	112	0	96	0	83	291
	PL82	95	0	105	0	103	303
	PL84	50	0	57	0	46	153
Portugal (PT)	PT11	818	0	456	0	332	1606
	PT15	80	0	60	0	37	177
	PT16	368	0	289	0	221	878
	PT17	663	0	256	0	231	1150
	PT18	75	0	109	0	103	287
Sweden (SE)	SE11	212	0	347	0	276	835
	SE12	247	0	220	0	192	659
	SE21	125	0	155	0	113	393
	SE22	196	0	205	0	185	586
	SE23	280	0	301	0	260	841
	SE31	128	0	137	0	129	394
	SE32	58	0	87	0	61	206
	SE33	78	0	102	0	98	278
Slovenia (SI)	SI03	761	0	687	0	669	2117
	SI04	507	0	439	0	494	1440
Slovakia (SK)	SK01	218	0	0	0	75	293
	SK02	594	0	0	0	360	954
	SK03	455	0	0	0	317	772
	SK04	539	0	0	0	313	852
United Kingdom (UK)	UKC	103	0	107	0	106	316
	UKD	257	0	230	0	226	713
	UKE	211	0	159	0	190	560
	UKF	166	0	159	0	149	474
	UKG	197	0	166	0	125	488
	UKH	204	0	188	0	199	591
	UKI	120	0	116	0	114	350
	UKJ	295	0	275	0	288	858
	UKK	177	0	180	0	187	544
	UKL	134	0	121	0	90	345
	UKM	227	0	192	0	169	588
	UKN	60	0	54	0	62	176
	Total	31879	890	29382	2395	32647	97193

ESS respondents were selected by means of strict probability samples of the resident populations aged 15 years and older at the country level. Respondents also provided information on their socio-demographic characteristics that we use as control measures in our model. We included a set of demographic variables such as age, gender, educational attainment, type of community the respondent resides in (urban versus rural), subjective income difficulties, and minority and citizenship status as controls. These are the factors found most commonly affecting attitudes to immigration ( Dražanová *et al.*, 2022). We restrict our sample to natives (defined as respondents born in the country where they were interviewed). We integrate the micro-attitudinal data from the ESS with contextual data at the regional and region-year level to capture the size and composition of the foreign-born population. These regional-level variables are gathered from various sources, particularly the European Labour Force Survey (EULFS) and the OECD’s database, which are described in more detail below.

The EULFS Data was received and processed through the European University Institute library following approval of research proposal number RPP 47/2021-LFby Eurostat Microdata Access Team. The data was stored and processed on a single designated computer located in a locked office of the EUI premises. Data analysis was conducted using Stata 15 and supported by the EULFS codebook and user guide. The EULFS microdata are anonymized according to anonymisation and aggregation criteria agreed between Eurostat and the National Statistical Institutes in order to enable Eurostat to make EU LFS microdata available to researchers.

### 3.1 Attitudes to immigration

The ESS survey instrument has been widely used by scholars to measure attitudes towards immigration ( Hainmueller & Hopkins, 2014). We distinguish between two types of attitudes to immigration in our analysis – a *ttitudes toward policy preferences regarding the level of immigration* and *the evaluation of the contribution and consequences of immigration on society.* These two dependent variables complement each other. The first one mostly deals with policy debates regarding immigration inflows and captures individuals’ preferences for the future. The second one represents opinions on whether immigration is beneficial to the community in the present.

Distinguishing between different types of attitudes to immigration has not always been the case in previous research. While these attitudes co-vary, they are not necessarily the same. For example, it is possible for a respondent to want to reduce the inflow of immigrants, but at the same time recognize their social and democratic rights once admitted. In this study, we specifically analyse attitudes toward allowing immigrants into the country and the perceptions of the effect of immigration. These are different, although strongly connected, dimensions of attitudes to immigration.

***3.1.1 Policy variable.*** Our policy dependent variable is a composite index that measures the overall willingness to allow only a few or many different types of immigrants into the country. Respondents were asked three questions: (1) To what extent do you think [country] should allow people of the same race/ethnic group as the majority to come and live here? (2) To what extent do you think [country] should allow people of different races/ethnic groups as the majority to come and live here? And (3) To what extent do you think [country] should allow people from the poorer countries outside Europe to come and live here? The answers are coded on a four-point scale ranging from (1) allowing many to come and live here to (4) allowing none. We created an average index and rescaled it so that it ranges from 0 to 1. ^3^ The original coding has been reversed so that higher numbers mean more positive attitudes. We included all respondents that have answered at least two of the three items comprising our dependent variable.

***3.1.2 Contribution variable.*** Our contribution dependent variable is a composite index that measures a person’s overall assessment of the impact of immigration on their society. Respondents were asked three questions: (1) Would you say it is generally bad or good for [country]’s economy that people come to live here from other countries? (2) Would you say that [country]’s cultural life is generally undermined or enriched by people coming to live here from other countries? and (3) Is [country] made a worse or a better place to live by people coming to live here from other countries? Answers are coded on an eleven-point scale where 0 is the most negative and 10 is the most positive reply. As with the policy variable, we created an average index ranging from 0 to 1, so that the two dependent variables are directly comparable. ^4^ We included all respondents that have answered at least two of the three items comprising our dependent variable.

Attitudes of immigration measured in a form of indices comprising several related questions have been widely used by scholars studying attitudes to immigration (see for example Davidov & Meuleman, 2012; Just & Anderson, 2015; Solheim, 2021 for the use of the policy index and Gorodzeisky & Semyonov, 2018; McLaren & Paterson, 2020 for the contribution index).

### 3.2 Regional migration data

We use repeated, cross-sectional data from the European Labour Force Survey (EULFS) to construct variables that capture the average and the short-term variations in the regional share of migrants at the NUT2 level. ^5^ These level and change variables are assigned to each ESS respondent based on the year they were interviewed and his or her region of residence. ^6^ Besides demographic information, the EULFS also reports the birthplace of each individual, distinguishing fifteen different regions of origin ^7^. We use all foreign-born individuals to compute a measure of the share of immigrants as a share of the total population at the regional level:

$$S_{r,t}^{s}=\frac{M_{r,t}^{s}}{Pop_{r,t}}$$

where M is the total stock of migrants in region r born in a foreign country, with skills (tertiary educated or not) and/or origin (Europe or non-European), or gender (male or female) in year t. ^8^ Thus, S represent that group of immigrants as a share of the total population. The average immigration variable is then constructed as:

$$avg_{r}^{s}=\frac{\sum_{t\in T}S_{r,t}^{s}}{\left|T\right|}$$

and represent the average share of immigrants from a given origin in region r over the time period T under investigation. For each region r, T corresponds to the period of time between the first and last year an individual was surveyed by the ESS in region r.

There are two ways to operationalize these regional demographics of interest, and we employ a longitudinal as well as a cross-sectional perspective for each (see methods section). Longitudinally, our main variable of interest captures how Europeans react to temporal shares of (non-)European foreign-born individuals that are below or above the regional average during the period of investigation.

Table 2 presents basic statistics for the variables we include in the model. Variables are averaged over the considered period at the individual level, region-year level and regional level. The average share of foreign-born living in the regions is 8.96 %, most of whom are of EU origin.

**Table 2.** Summary statistics.

	Full sample				
	N	Mean	S.D.	Min	Max
Contribution	94110	0.505	0.217	0	1
Policy	94474	0.529	0.273	0	1
***Individual level***					
age	96919	49.973	18.799	14	104
university	94909	0.273	0.445	0	1
tertiary without degree	94909	0.054	0.225	0	1
Upper secondary	94909	0.389	0.488	0	1
Lower secondary	94909	0.174	0.379	0	1
female	97146	0.53	0.499	0	1
Living in urban area	97193	0.294	0.456	0	1
Income difficulty	96052	0.235	0.424	0	1
minority	95967	0.091	0.287	0	1
non citizen	97137	0.003	0.056	0	1
***Region-year level***					
Change in share of foreign-born	580	-0.059	1.257	-7.037	5.439
Change in share of foreign-born from Europe	580	-0.020	0.683	-3.467	3.62
Change in share of foreign-born outside Europe	580	-0.039	0.874	-4.991	4.011
***Regional level***					
Share of foreign-born	183	8.966	6.702	0.129	42.499
Share of foreign-born from Europe	183	5.183	4.094	0.129	21.778
Share of foreign-born outside Europe	183	3.780	3.829	0	22.353
GDP per capita (PPS)	177	24674	9008.3	7007	57365
% unemployed 15+	181	10.152	4.808	2.6	31.9
Population density	177	361.415	939.455	3.3	6957.2

### 3.3 Empirical strategy

As an empirical strategy, we employ random effects multilevel modelling tailored to the structure of repeated cross-sectional data that allows us to decompose the variance of the outcome (immigration attitudes) into a within- and between- region part ( Bell *et al.*, 2019; Fairbrother, 2014). These models are four-level hierarchical linear models, with individuals nested in region-years nested in regions nested in countries respectively ( Schmidt-Catran & Fairbrother, 2016).

The four-level random intercept multilevel models are estimated using restricted maximum likelihood (reml).

Our final four-level model ^9^ is defined as:

$${\text{Y}}_{\text{ijkc}}=\beta_{0\text{ijkc}}+\beta_{1}\;{\text{X}}_{\text{ijkc}}+\beta_{2}\;S_{jkc}^{s}+\beta_{3\;}avg_{kc}^{s}+\beta_{4}\;{\text{W}}_{\text{jkc}}+\beta_{5}\;{\text{Z}}_{\text{c}}+f_{\text{c}}+\mu_{\text{kc}}+{\text{v}}_{\text{jkc}}+{\text{e}}_{\text{ijkc}}$$

where, within each region-year *j*, region *k* and country *c*, respondents' attitudes to immigration (Y) are a function of their individual characteristics (vector X), the demeaned version of the variable capturing the annual share of immigrants S, whether at the aggregate level or distinguishing between their origin, the average regional share of immigrants *avg* – also origin - over the whole time period considered, region-year characteristics (vector W) and Western/Eastern country-year binary combinations (vector Z). β _0ijkc _is the mean of attitudes to immigration of individuals in region-year *j*, region *k*, and country *c*, β _1_ is the level-1 fixed effects, β _2_ and β _4 _are the level-2 fixed effects, β _3 _is the level-3 fixed effects and β _5_ are the level-4 fixed effects. In the random part of the model *f _c_* is the residual random effect of country *c,* μ _kc_ is the residual random effect of region *k,* ν _jkc_ is the residual random effect of region-year *j* and e _ijkc _is the random individual variation. The random effects μ _kc_, ν _jkc_ and *f*_c _are assumed normally distributed with mean 0 and variance τ _μ_, τ _ν_ and τ _f_ respectively.

A series of individual sociodemographic controls are included. We control for a person’s age (in years), gender (female), and education (four categories with less than lower secondary as reference). Dummy variables are included to control for individuals who live in urban areas (urban area=1) and report having income difficulties (income difficulty=1). We also include a minority dummy for respondents whose at least one parent was born outside of the country and/or are part of an ethnic minority (minority=1). Finally, we also control for respondents’ citizenship status (non-citizens=1), since our sample is restricted to respondents who were born in the country but might not be citizens.

The demeaned variable for immigration *S* yields within regional effects or, in other words, the longitudinal within-region change component (WE) (previously referred to as inflows or short-term variations) for each observation at region-year, while the mean variable *avg* captures cross-sectional between regional effects (BE). The advantage of this four-level multilevel model is that it distinguishes between-regional effects and within-regional change while controlling for compositional differences at the individual level (see Fairbrother, 2014). Within-effects automatically control for all regional characteristics that are time-invariant and are not afflicted by omitted variable bias due to any time-constant aspects on the regional level such as stable differences in political, historical or legal factors. Between effects are, in turn, based only on time-stable differences between regions.

Apart from controlling for within and between regional effects, we also control for clustering at the country level since possible clustering at the country level might still occur ( Schmidt-Catran & Fairbrother, 2016). We employ a Western/Eastern country-year dummy to model a general geography-time trend.

We also collect data about GDP and unemployment rate from the OECD database and Eurostat to use as controls for time-varying differences across regions that could influence individuals’ attitudes to immigration. A contextual variable regarding regional population density was also added to the model. Since these are not of our primary interest, only between-region (and not also within-region) macro indicators are included. A similar approach has been used by McLaren (2012) and Jeannet (2020) for country-level controls.

We do not control specifically for any country-level characteristics apart from countries being either part of Western or Central and Eastern Europe. Nevertheless, we assume that individuals from the same country are significantly more similar in their attitudes to immigration than individuals from different countries. This is confirmed by the likelihood ratio (LR) tests comparing a three-level model (individuals nested within region-year and region) to a four-level model (individuals nested within region-year, region and country) ( *χ*_1_^2^ = 174.94, *p* < 0.001 for contribution and *χ*_1_^2^ = 223.85, *p* < 0.001 for policy). Thus, respondents from the same country are significantly more alike in their attitudes to immigration than respondents from different countries.

Clustering at the country level also distinguishes our analysis from the one conducted by Hale Williams and Chasapopoulos (2019). While Hale Williams and Chasapopoulos (2019) employ multilevel modelling, they do not cluster regions within countries. As shown in Table 3 in the two null models, when including countries as a level-4 cluster, they represent the most important clustering factors on immigration attitudes and the regional variation becomes negligible.

**Table 3.** Multilevel regressions of attitudes toward immigrants, individual controls only.

	Attitude Toward Immigrants´ Contribution				Attitude Toward Immigration Policy			
	Model 0		Model 1		Model 0		Model 1	
	β	S.E.	β	S.E.	β	S.E.	β	S.E.
*Individual level effects*								
age			0.0007***	(0.000)			0.002***	(0.0001)
university			0.131***	(0.0026)			0.146***	(0.0032)
tertiary without degree			0.069***	(0.0036)			0.081***	(0.0045)
Upper secondary			0.047***	(0.0025)			0.057***	(0.0031)
Lower secondary			0.030***	(0.0027)			0.042***	(0.0033)
female			-0.001	(0.0013)			0.006***	(0.0016)
Living in urban area			0.015***	(0.0015)			0.018***	(0.0019)
Income difficulty			-0.050***	(0.0017)			-0.048***	(0.0021)
minority			0.035***	(0.0023)			0.039***	(0.0029)
non citizen			0.015	(0.012)			0.010	(0.0149)
Intercept	0.505***	(0.014)	0.482***	(0.013)	0.533***	(0.019)	0.545***	(0.018)
*Random effects*								
country	0.004	(0.001)	0.003	(0.0011)	0.007	(0.002)	0.0064	(0.002)
region	0.0005	(0.001)	0.002	(0.0001)	1.30e-14	(4.71e-13)	3.16e-17	(1.64e-13)
Region-year	0.0018	(0.001)	0.002	(0.0001)	0.004	(0.0003)	0.0039	(0.0002)
Individual	0.412	(0.002)	0.037	(0.0001)	0.062	(0.0002)	0.0578	(0.0002)
N respondents	94 110		89 634		94 474		90 036	
N countries	21		21		21		21	
N regions	183		183		183		183	
N region-years	580		574		580		574	

It is important to incorporate four-level structures in the models when they arise in the data and lead the higher-level clusters to differ substantially from one another on the response variable. Fitting models with a lower number of levels to data with, in fact, more hierarchical clusters could lead to misattributing response variation to only the included levels. This in turn may lead to drawing misleading conclusions about the relative importance of different sources of influence on the response.

Table 3 shows two null (or so-called “empty”) models in order to partition the variance of our two dependent variables of interest across the four levels. This model provides information on the variance components of immigration attitudes at each level of analysis (Level 1 - individual, Level 2 – region-year, Level 3 – region and Level 4 - country). It includes only an intercept, region-year random effects, region random effects, country random effects and an individual level residual error term. The overall mean attitude toward immigrants´ contribution across all countries, all regions, all region-years and all respondents is estimated to be 0.505 on a scale of 0–1, whereas the overall mean attitude toward immigration policy across countries, all regions, all region-years and all respondents is estimated to be 0.533 on a scale 0–1. ^10^

The null model shows that 86.6 % of the variation in attitudes toward immigrants’ contribution lies between individuals within region-years, 3.8 % lies between region-years within regions, 1 % lies between regions within countries and 8.6 % lies between countries. On the other hand, 83.6 % of the variation in attitudes toward immigration policy lies between individuals within region-years, 6 % lies between region-years within regions and 10.4 % lies between countries. There is no variation between regions within countries for attitudes toward immigration policy. However, as we are interested mostly in the effect of region-year variations, this shall not pose a problem for our models. At first, 3.8 and 6 % might seem small, but the longitudinal variance excludes all variation that is due to time-invariant idiosyncrasies between regions as well as between countries. The resulting within (WE) effects in further models thus exclude the impact of all-time stable confounding aspects, which is an advantage of our modelling strategy compared to usual cross-sectional estimates.

Most of the variation in attitudes to immigration is found at the individual level, which is consistent with previous literature regarding differences in immigration attitudes. However, there is also a modest variation at the country, region (for attitude toward immigrants’ contribution) and region-year level, thus justifying a multilevel approach.

## 4. Results

Our baseline analysis captures how public opinion varies with the average and short-term variations in the regional share of foreign-born individuals over the 2010–2019 period. All models presented hereafter include individual controls mentioned above as well as regional, time-varying variables that are likely to influence public opinion towards immigration over time such as GDP, unemployment, and the density of population.

**Figure 1.**
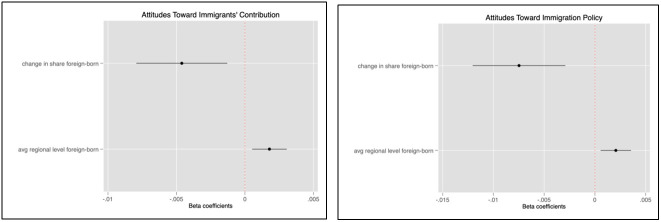
Multi-level coefficients for regional shares of foreign-born with confidence intervals.

Figure 1 presents the results for the full sample estimated using multi-level restricted likelihood and four levels of nesting (country, region, region-year and individuals). By including both the average level of foreign-borns’ presence and the short-term variations due to migration pressure measured as deviations from this mean (inflows or outflows, see section 3.2), we are able to disentangle between the channels that are driving the relationship between the size of immigration and public attitudes. In particular, while the *avg* variable measures variations in opinion on immigration that are imputable to differences between regions, the *change* variable captures the reaction of individuals with respect to within-region changes in the share of immigrants over time.

Our results in Figure 1 and Table 4 indicate that on aggregate, the share of immigrants in a given region is associated with more positive attitudes towards immigrants in matters of both migration policy and individual feelings about immigrants’ contribution. ^11^ This result is in line with the contact theory, which posits that prolonged interaction with high levels of immigration at the local level increases the incidence of contact between natives and newcomers and therefore reduces prejudice and the perception of threat in the long run. The coefficient measuring attitudes towards immigrants’ contribution suggests that, *ceteris paribus*, a 10 % increase in the average share of immigrants (in absolute terms) across regions is associated with an increase in positive attitudes by 1.8 percentage points on the contribution scale. ^12^ This effect is slightly larger (2.1 %) and still very significant when respondents are asked about their opinion on migration policy. Moreover, our analysis suggests a negative association between a short-term increase in the share of immigrants and attitudes towards immigration. Controlling for differences in the average share of immigrants across European regions, we find that a 10 % increase in the share of immigrants is associated with a decrease in support for allowing more immigrants by 7.5 % within a given region. This negative association (- 4.6 %) is also significant for attitudes towards migrants’ contribution.

**Table 4.** Multi-level estimation results, total immigration - Attitudes toward immigrants’ contribution.

	β	S.E.	β	S.E.	β	S.E.	β	S.E.
age	-0.0007 ***	(0.00003)	-0.0017 ***	(0.0004)	-0.0007 ***	(3.71e-05)	-0.0007 ***	(3.69e-05)
university	0.1316 ***	(0.0263)	0.146 ***	(0.0032)	0.131 ***	(0.00263)	0.131 ***	(0.00262)
tertiary without degree	0.0694 ***	(0.0367)	0.0808 ***	(0.0045)	0.0691 ***	(0.00367)	0.0691 ***	(0.00366)
Upper secondary	0.0472 ***	(0.0257)	0.057 ***	(0.003)	0.0470 ***	(0.00257)	0.0472 ***	(0.00257)
Lower secondary	0.0307 ***	(0.0277)	0.0416 ***	(0.003)	0.0305 ***	(0.00277)	0.0303 ***	(0.00276)
female	-0.0012	(0.0132)	0.006 ***	(0.0131)	-0.00117	(0.00132)	-0.00116	(0.00131)
Living in urban area	0.0149 ***	(0.0159)	0.0181 ***	(0.0019)	0.0151 ***	(0.00159)	0.0154 ***	(0.00158)
Income difficulty	-0.0497 ***	(0.0172)	-0.048 ***	(0.002)	-0.0498 ***	(0.00172)	-0.0502 ***	(0.00171)
minority	0.0347 ***	(0.0237)	0.0389 ***	(0.0029)	0.0347 ***	(0.00237)	0.0347 ***	(0.00236)
non citizen	0.0156	(0.121)	0.0102	(0.0149)	0.0152	(0.0121)	0.0150	(0.0121)
change in share foreign- born	-0.0046 **	(0.0016)	-0.006 **	(0.0023)				
avg regional level foreign- born	0.0017 **	(0.0067)	0.0020 ***	(0.0005)				
change in share European foreign-born					0.00380	(0.00260)	0.00387	(0.00262)
avg regional level European foreign-born					0.00364 ***	(0.00110)	0.0043 ***	(0.00106)
change in share non- European foreign-born					-0.0110 ***	(0.00226)	-0.0102 ***	(0.00228)
avg regional level non- European foreign-born					0.000297	(0.00115)	-0.0138	(0.0983)
regional gdp per capita	7.29e-06	(4.29e-06)			6.57e-07	(3.77e-07)		
regional unemployment	-0.006	(0.0072)			-0.00147	(0.00075)		
regional density	-5.45e-05	(3.93e-05)			-4.73e-06	(3.82e-06)		
*Random effects*								
country	0.003	0.001	0.005	0.001	0.0034	(0.0012)	0.032	(0.001)
region	0.0003	0.001	0.0003	8.23e-06	0.0003	(0.00009)	0.0003	(0.00009)
Region-year	0.007	0.001	0.0016	0.00019	0.0007	(0.00008)	0.0007	(0.00008)
Individual	0.0379	0.001	0.057	0.0004	0.0379	(0.0001)	0.037	(0.0001)
Intercept	0.428 ***	(0.0230)	0.560 ***	(0.0287)	0.472 ***	(0.257)	0.462 ***	(0.022
N respondents	89,001		89,634		89,001		89,634	
N countries	21		21		21		21	
N regions	182		182		182		183	
N region-years	566		566		566		574	

All full sample models control for Western/Eastern Europe-year dummy variables. *** p<0.001, ** p<0.01, * p<0.05

These results are in line with the findings of Coenders and Scheepers (2008) and Hopkins (2010), who suggest that negative reactions to immigrants are more likely to occur in response to competition from recent foreign arrivals, rather than existing diversity.

Recent evidence suggests that European natives’ attitudes to the arrival of foreign-born migrants may vary based on the composition of migration flows (see for instance Dražanová and Geddes, (2022) on the differences in European public opinion towards Syrian and Ukrainian refugees). In this regard, we test whether the origin of immigrants affects the sign and magnitude of the association between attitudes and immigration.

**Figure 2.**
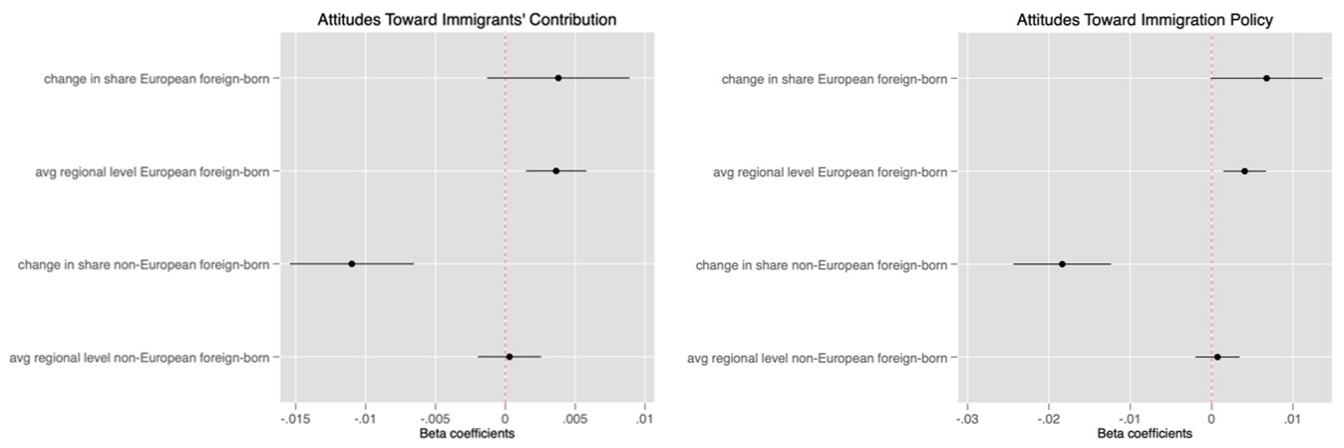
Multi-level coefficients of shares of foreign-born by region of origin with confidence intervals.

Figure 2 and Table 5 distinguish between the flows of European and non-European immigrants. In line with the existing literature documenting a negative bias towards immigrants that are ethnically and culturally more distant ( Murard (2017); Moriconi *et al.* (2019)), we find that a similar bias largely applies to non-European immigrants for both policy and contribution dependent variables: The coefficients are negative and statistically significant, suggesting that a 10 % increase in the share of non-European immigrants at the regional level is associated with respectively an 11 % and 19 % decrease in natives’ opinion about the contribution of immigrants and support for immigration. Moreover, we find no significant negative correlation between the arrival of European immigrants and natives’ attitudes. Instead, these coefficients – which are statistically significant at the 10% level - point towards a positive relationship. Likewise, the coefficients of the *avg* variable suggest that views about immigration are significantly more positive in regions that host a higher share of EU immigrants.

**Table 5.** Multi-level estimation results, total immigration - Immigration policy.

	β	S.E.	β	S.E.	β	S.E.	β	S.E.
age	-0.002 ***	(4.56e-05)	-0.0017 ***	(4.54e-05)	-0.0017 ***	(4.56e-05)	-0.0017 ***	(4.54e-05)
university	0.146 ***	(0.00323)	0.146 ***	(0.00322)	0.146 ***	(0.00323)	0.146 ***	(0.00322)
tertiary without degree	0.080 ***	(0.00453)	0.0809 ***	(0.00452)	0.0810 ***	(0.00453)	0.0810 ***	(0.00452)
Upper secondary	0.057 ***	(0.00315)	0.0578 ***	(0.00315)	0.0577 ***	(0.00315)	0.0579 ***	(0.00315)
Lower secondary	0.041 ***	(0.00339)	0.0416 ***	(0.00338)	0.0419 ***	(0.00339)	0.0417 ***	(0.00338)
female	0.006 ***	(0.00163)	0.0061 ***	(0.00162)	0.0062 ***	(0.00163)	0.0061 ***	(0.00162)
Living in urban area	0.018 ***	(0.00196)	0.0181 ***	(0.00195)	0.0181 ***	(0.00196)	0.0182 ***	(0.00195)
Income difficulty	-0.048 ***	(0.00211)	-0.0489 ***	(0.00210)	-0.0486 ***	(0.00211)	-0.0490 ***	(0.00210)
minority	0.039 ***	(0.00291)	0.0390 ***	(0.00290)	0.0391 ***	(0.00291)	0.0390 ***	(0.00290)
non citizen	0.010	(0.0149)	0.0102	(0.0149)	0.0104	(0.0149)	0.0100	(0.0149)
change in share foreign- born	-0.007 **	(0.00233)	-0.0068 **	(0.00233)				
avg regional level foreign- born	0.002 **	(0.0007)	0.0020 ***	(0.00055)				
change in share European foreign-born					0.00676	(0.00352)	0.00665	(0.00353)
avg regional level European foreign-born					0.00407 **	(0.00133)	0.0048 ***	(0.00128)
change in share non- European foreign-born					-0.0184 ***	(0.00306)	-0.0172 ***	(0.00307)
avg regional level non- European foreign-born					0.000726	(0.00138)	-0.000275	(0.00118)
regional gdp per capita	8.10e-07	(4.73e-07)			7.34e-07	(4.71e-07)		
regional unemployment	-0.00156	(0.00095)			-0.00184	(0.000954)		
regional density	-7.42e-06	(4.53e-06)			-7.80e-06	(4.63e-06)		
*Random effects*								
country	0.005	(0.001)	0.005	(0.001)	0.005	(0.002)	0.0057	(0.001)
region	0.0002	(0.001)	0.0003	(8.23e-06)	0.003	(0.0001)	0.0004	(0.0001)
Region-year	0.016	(0.001)	0.001	(0.0001)	0.0014	(0.0001)	0.0014	(0.0001)
Individual	0.057	(0.0002)	0.057	(0.0004)	0.0578	(0.0002)	0.0578	(0.0002)
Intercept	0.562 ***	(0.032)	0.560 ***	(0.028)	0.562 ***	(0.0334)	0.552 ***	(0.029)
N respondents	89,378		90,036		89,378		90,036	
N countries	21		21		21		21	
N countries	182		183		182		183	
N region-years	566		574		566		574	

All full sample models control for Western/Eastern Europe-year dummy variables. *** p<0.001, ** p<0.01, * p<0.05

These results suggest that the negative association between changes in foreign-born immigration and attitudes is entirely driven by the arrival of immigrants from outside the European Union. It is therefore possible that the negative reactions to immigrants in response to competition from recent foreign arrivals documented in the literature only materialize when those migrants are from a different origin, or ethnically distant from natives, to the extent that origin can be regarded as valid cue for ethnic proximity.

In contrast, the positive association between the historical presence of migrants and public opinion about immigration presented in Figure 1 is driven by the arrival of migrants from within the European Union, which could be interpreted as evidence that the contact hypothesis only has traction when migrants are ethnically closer to natives.

Put together, these findings indicate that the temporal dynamics of attitudes to immigration vary with the origin of immigrants. Only the presence of EU immigrants is significantly correlated with changes in the perception and political preferences of public opinion regarding immigration in the long term (historical presence). Investigating what lies behind this pattern is beyond the scope of our analysis, but further investigation in this direction is necessary.

## 5. Discussion and conclusion

In recent years, European countries have experienced a surge in migration flows and public resentment against immigrants among their domestic population.

This paper proposes a novel empirical design to study how public attitudes to immigration reacted to increased migration pressure across European regions over the past decade. We explore the nature of this relationship beyond cross-region differences and focus our attention on the predictive power of within-region, short-term migration flows. Controlling for important individual cofounders and contextual drivers of attitudes to immigration, we examine how variations in migration pressure correlate with public opinion towards natives’ support for immigration and their views of immigrants’ contribution to their destination country. Our analysis is informed by theories of economic competition between natives and immigrants, cultural backlash, and the contact hypothesis, which are all part of the canonical framework developed by social scientists to study public opinion towards immigration.

At the aggregate level, across all European regions contained in the sample, our findings indicate that immigration is positively correlated with natives’ attitudes regarding migration policy and opinions about immigrants’ contribution, in line with the contact hypothesis. Further analysis concerning the composition of migration flows is consistent with theories of economic and ethnic competition. In particular, we find that inflows of EU-origin are positively correlated with natives’ attitudes.

We must stress that our empirical design does not permit us to make causal predictions about the role played by immigrant inflows on public opinion and predict with certainty the risks of tensions that may arise from increased migration pressure. Indeed, exploring the causal relationship between migration flows and attitudes towards immigration would require accounting for endogeneity biases such as the self-selection of migrants into areas with better economic conditions or where natives happen to be less hostile to immigrants. For instance, European immigrants are likely to face fewer constraints in the choice of destination when migrating because of their greater freedom of movement. To the extent that further immigration tends to polarize attitudes to immigration, whereby regions with more positive (resp. negative) opinions tend to become more positive (resp. negative) with the arrival of new immigrants, the correlation found in our study could thus be artificially inflated.

Finally, it is possible that natives with the most negative attitudes simply move out of regions receiving more immigrants, and that our results are driven by a crowding-out effect (see Dustmann & Preston, 2001).

That said, we believe our analysis informs the current political debate about the consequences of short-term migration flows on public attitudes to immigration in several ways. First, our study of regional migration flows furthers our understanding of how European public opinion may respond to local migration and can help policymakers and practitioners anticipate potential risks of tensions as a result of future migration. That said, further research remains necessary to investigate whether migration pressure has a direct and causal impact on attitudes to immigration.

## Ethical approval and consent

Ethical approval and consent were not required.

## Data Availability

ESS Data used in this study are extracted from round 4, 5, 6, 7 and 8 of the European social survey. This data is publicly available from the European Social Survey (ESS) data portal: https://doi.org/10.21338/NSD-ESS-CUMULATIVE. Access to microdata from the European Union Labour Force Survey (EU-LFS) is granted for legitimate research purposes only. To protect the anonymity of respondents (persons, organisations), the access to microdata is restricted and requires filing a research proposal to the Eurostat Microdata Access Team. A guide for how to apply for dataset access is available at: https://ec.europa.eu/eurostat/documents/203647/771732/How_to_apply_for_microdata_access.pdf Data from the OECD is publicly available from the following link: https://stats.oecd.org/
