# Supplementary material for: Attitudes toward immigration in Europe: Cross-regional differences
Source: Open Res Eur. 2023 Apr 28;3:66. [Version 1] doi: 10.12688/openreseurope.15691.1 (PMC10445868; doi:10.12688/openreseurope.15691.1)
Supplement: Peer review history

# Peer review: Reviewer response for version 1

**DOI:** 10.21956/openreseurope.16952.r31418

**Published:** 2023-06-16

**Authors:** Francisco Rowe

**Affiliations:** 1 Department of Geography and Planning, University of Liverpool, Liverpool, England, UK

The paper is well structured, and I think It makes a contribution to an area of much needed attention; that is, understanding geographic variations in attitudes towards immigration. There is a consolidated body of evidence of geographic variations in relation to immigration sentiment. Yet, limited progress has been made on advancing empirical research and conceptual thinking in understanding how this occurs - how we find such wide variations within a country. Hence, I think the paper is a welcome contribution to the literature on attitudes toward immigration, but I have some major concerns regarding the analysis and conceptual ideas being tested.

 1. I would like to raise questions about the geographical scale of the analysis. I agree on the importance of considering and understanding geographical variations in attitudes towards migration. I think that is a valuable endeavour, but I am not persuaded about the geographic scale of analysis. What is the rationale of the analysis to focus on a regional scale? What is the conceptual link between immigration sentiment and the regional net rate of foreign-born population? Most migration theories would consider the proximate environments where people live (i.e. neighbourhoods, cities, towns) to be the relevant unit of analysis as local contextual factors operate at this level. It is at this level at which such factors are argued to operate influencing individual perceptions, and at which face-to-face interactions between migrants and the local born occur. The geographic unit used in the analysis seems to have been a function of data availability (which is fine), but is it the appropriate geography for analysis? What are the hypotheses that are and can be tested at the level of geography used in the analysis?

 2. Definition of the gap in the literature. I think the gap in the literature that the paper is seeking to fill should be more clearly defined. The paper seeks to differentiate itself from existing literature by focusing on how short-term changes in the share of migrant population may influence public opinion on immigration and developing an argument that there is limited empirical research examining this changes at a regional level. While I agree on the lack of research seeking to understand public opinion towards migration at local scales and their temporal changes, the paper should explicitly state why analysing this is conceptually relevant.

 3. The study emphasises the importance of considering the temporal dynamics of attitudes towards immigration, but it does not consider research based on digital footprint data capturing how immigration sentiment is highly responsive to contemporary events. Given that this is one of the main arguments in the conclusion of the paper, I feel this body of work should be appropriately considered in the introduction, background, and discussion and conclusion sections. Key pieces that I should should be included are: the work by Rowe *et al* on immigration sentiment and recent reports by the IOM and JRC - see relevant references below. I see this work being particularly relevant because news and representations about migrants are likely to be more influential on shaping public opinion on immigration than numbers of migrants per se. But only the latter is the focus of the paper.

- Rowe, F., Mahony, M., Graells-Garrido, E., Rango, M. and Sievers, N., 2021. Using Twitter to track immigration sentiment during early stages of the COVID-19 pandemic. Data & Policy, 3, p.e36 ^1^.
- Rowe, F., Mahony, M., Graells-Garrido, E., Rango, M. and Sievers, N., 2021. Using Twitter Data to Monitor Immigration Sentiment. Harnessing Data Innovation for Migration Policy: A Handbook for Practitioners. International Organization for Migration (IOM). IOM, Geneva. pp. 104-119 ^2^.
- Freire-Vidal, Y., Graells-Garrido, E. and Rowe, F., 2021. A framework to understand attitudes towards immigration through Twitter. Applied Sciences, 11(20), p.9689 ^3^.
- International Organization for Migration (IOM), 2023. Harnessing Data Innovation for Migration Policy: A Handbook for Practitioners. IOM, Geneva ^4^.
- Bosco, C., Grubanov-Boskovic, S., Iacus, S., Minora, U., Sermi, F. and Spyratos, S., Data Innovation in Demography, Migration and Human Mobility, EUR 30907 EN, Publications Office of the European Union, Luxembourg, 2022, ISBN 978-92-76-46702-1, doi:10.2760/958409, JRC127369 ^5^.

4. The introduction describes related work but it is unclear how the work discussed is related to the current paper. Elaboration on this is needed, specifically describe how the current paper fits within the broader literature.

 5. The background is relatively short to other sections. Particularly important would be to include a more comprehensive discussion on the relationship between attitude formation and the size and speed of local immigrant communities. I feel that the current discussion should be expanded.

 6. Treatment of time. The methodology does not explicitly describe how time is being modelled. Are independent variables lagged? Or are they on the same time period as the dependent variable? Please explain how this is done and why, as well as any issues readers should be aware with the approach taken.

 7. Related to the previous point, a variable labelled “average immigration variable” which is constructed using all years as the denominator i.e. 2012-16 or 18. Does this mean sense if the dependent variable is measured at different points in time. For example, how do we conceptually explain the relationship between a dependent variable measured in 2010 and an independent variable x measured in 2018?

 8. The paper describes the application of a nested model using 4 levels. However, some of the levels used are not mutually exclusive pointing to a potential model specification issues. A cross-classified specification seems more appropriate in this case. Please amend the model specification appropriately i.e. please justify your model specification, or amend the model.

 9. The type of model estimated is not stated in the description of the methodology. Was it a linear or a logistic regression? The fact that the dependent variable is bounded to 0 and 1 imposes some challenges. Please describe the model estimated and how you answer the underlining challenges of modelling a variable bounded between 0 and 1. Does the model used produce predicted values of the dependent variable beyond this range?

 10. P11. Discusses the results from the multilevel model reported in Table 3. The paragraph on the second column discusses the variability in the dependent variable explained by the model at various levels. I was unable to reproduce the results discussed with the data provided in Table 3. How these results were produced should be explained in the text so readers are able to replicate the reported numbers.

 11. I could not find details about the software code used and how readers would go about processing the data to replicate the analysis. Would encourage authors to share their code through open repositories so others can replicate the analysis and reuse it on their own data.

If applicable, is the statistical analysis and its interpretation appropriate?

Partly

Is the study design appropriate and is the work technically sound?

Partly

Is the work clearly and accurately presented and does it engage with the current literature?

Partly

Are the conclusions drawn adequately supported by the results?

Partly

Are sufficient details of methods and analysis provided to allow replication by others?

No

Are all the source data and materials underlying the results available?

Partly

Reviewer Expertise:

Migration, human mobility, geographic data science, spatial inequality

I confirm that I have read this submission and believe that I have an appropriate level of expertise to confirm that it is of an acceptable scientific standard, however I have significant reservations, as outlined above.

## References

[1] : Using Twitter to track immigration sentiment during early stages of the COVID-19 pandemic. *Data & Policy* .2021;3: 10.1017/dap.2021.38 10.1017/dap.2021.38

[2] : Using Twitter Data to Monitor Immigration Sentiment. Practitioners’ Guidebook. International Organization for Migration. *United Nations. Berlin, Germany* .2021; Reference source

[3] : A Framework to Understand Attitudes towards Immigration through Twitter. *Applied Sciences* .2021;11(20) : 10.3390/app11209689 10.3390/app11209689

[4] : Harnessing Data Innovation for Migration Policy: A Handbook for Practitioners. *IOM, GENEVA.* .2023; Reference source

[5] : Accessibility of DNA in condensed chromatin to nuclease digestion. *Nature* .1976;262(5568) : 10.1038/262516a0 516-9 10.1038/262516a0 958409

# Peer review: Reviewer response for version 1

**DOI:** 10.21956/openreseurope.16952.r32203

**Published:** 2023-06-12

**Authors:** Cengiz Erisen

**Affiliations:** 1 Department of Political Science and International Relations, Yeditepe University, Istanbul, Turkey

I really enjoyed reading the article. The authors attempt to test the connection between attitudes toward migration and regional influences (i.e., foreign-born share in the region and change in that indicator). The authors tease out the differences between European and non-European foreign-born migrant share in the region and find that Europeans are sensitive to the origin of the migrants. Using the large-n ESS data and random effects multi-level analysis, authors provide an observational test of their main expectation. All in all, there is much to like in this work, and I support the indexing of this work after some minor revisions.

 First, the authors repeatedly refer to the concept of “regional differences” at the front end of the paper. However, this is unclear because they use different wording at each time. First, they refer to “migration flows” on page 3, and in the same paragraph, they refer to the “arrival of migrants.” Later, on page 4, they refer to “immigrants’ historical presence” and then use “migration inflows.” These all refer to the same concept of “contextual migrant effects in the region,” but we see differing versions. So, I recommend making this clear in this regard.      

 Second, regarding the empirical analysis, I have a few comments: 1. Table 1 is unnecessary and can be carried over to the appendix; 2. Tables 3 and 4 are also unnecessary. The main results of these tables are presented in figures, which very nicely present the main effects; 3. In the three tables reporting the primary empirical analysis, almost every individual-level indicator is significant. As the authors focus on the regional effects, they omit the individual-level effects. With that, how can we make sense of a model where all of the individual-level indicators are significant on the DV, and yet the regional effects matter the most? These individual-level indicators require discussion and connecting with the overall empirical approach.

 Third, the authors suggest that according to the contact hypothesis, Europeans favor foreign-born from Europe migrants (e.g., Ukrainians), as opposed to foreign-born outside Europe (e.g., Syrians). I don’t think that we can separate the expectations of the contact hypothesis on the basis of foreign-born Europe vs. non-Europe. For the contact hypothesis to work, specific requirements must be fulfilled (regardless of the migrant's birth location), which we don’t see being tested here. What the authors find here is closer to social identity theory (SIT) preferences, whereby the host country members favor in-group migrants instead of out-group migrants. The authors can find more discussion about this subject in Erisen & Kentmen-Cin (2017) ^1^ and Kentmen-Cin and Erisen (2017) ^2^. So, I recommend that the authors reconsider their inferences and relate their argument to the relevant literature.        

 Minor issue: authors use too many self-pronouns and third-person pronouns for the authors to whose work they refer. Referring to the authors by name or calling them “the author(s)” would read better.

 Again, I enjoyed reading this manuscript and support its indexing after minor revisions. I believe that the work will make a fine contribution about the regional/contextual effects of migrants in individual-level preferences on migration policies.

If applicable, is the statistical analysis and its interpretation appropriate?

Yes

Is the study design appropriate and is the work technically sound?

Yes

Is the work clearly and accurately presented and does it engage with the current literature?

Yes

Are the conclusions drawn adequately supported by the results?

Yes

Are sufficient details of methods and analysis provided to allow replication by others?

Yes

Are all the source data and materials underlying the results available?

Yes

Reviewer Expertise:

Comparative Political Behavior, Political Psychology, Public Opinion research

I confirm that I have read this submission and believe that I have an appropriate level of expertise to confirm that it is of an acceptable scientific standard.

## References

[1] : Tolerance and perceived threat toward Muslim immigrants in Germany and the Netherlands. *European Union Politics* .2017;18(1) : 10.1177/1465116516675979 73-97 10.1177/1465116516675979

[2] : Anti-immigration attitudes and the opposition to European integration: A critical assessment. *European Union Politics* .2017;18(1) : 10.1177/1465116516680762 3-25 10.1177/1465116516680762

# Peer review: Reviewer response for version 1

**DOI:** 10.21956/openreseurope.16952.r31419

**Published:** 2023-05-24

**Authors:** Linda Basile

**Affiliations:** 1 Department of Social, Political and Cognitive Sciences, University of Siena, Siena, Italy

The article explores the impact of region-level dynamics on individual attitudes toward immigration. In particular, the authors focus on both the short and long-term dynamics of migration at the regional level. I appreciate the choice to focus on the regional level, which is a rather under looked perspective for analyses of public preferences and that is why I support this submission.

 Nonetheless, I would like to advance a few comments that might help the authors to improve the manuscript. I mention them not in order of relevance, but as they appear in the manuscript.

 1. The research fully engages in a complete, yet concise review of the main literature, which can be easily summarised in the three key theories of economic competition, cultural threat, and contact theory. Indeed, this theoretical background fully justifies the results. However, the authors do not present hypotheses based on theories. Advancing hypotheses would help better identify the dynamics that could explain the different effects of short and long-term migration. In particular, economic/cultural theory would explain the negative attitudes following a short-term increase in migration, whilst the contact theory would explain the effect of long-term migration.

 2. At p.5 I did not understand very well why the authors restrict the sample to natives rather than including the whole sample and probably controlling for the effect of being native or not. The choice can be reasonable but need to be better justified. Also, they should report the share of non-natives that were included in the original sample.

 3. On p. 7 the authors mention data from both EULFS and OECD, but in the end, they refer only to EULFS on p.8, please clarify on the use of OECD data. 

 4. The description of the main independent variable on p. 8 refers to migrants' skills in the formula used to calculate the share of migrants, yet it is not clear how this data is calculated and whether it is used in the analyses (it seems not). 

 5. I would suggest removing Table 1 (it is enough to mention in the paper the average number of respondents per region, plus the regions with lower and higher numbers of respondents); rather, I would include a table or some nice descriptives about the regional distribution of migrants, both from and outside the EU. This could also be done through a map that could help better visualise regional differences in patterns of migration (indeed, I suspect that these variations tend to cluster by countries, but there might be differences, for example, between some coastal regions in southern countries and others more internal).

 6. The empty models and the ICC (I think the percentages reported on p.11 refer to it, and this should be clarified) confirm a limited impact of regional-level effect, whilst attitudes towards migration tend to hinge upon individual characteristics. This is in line with some research I am also conducting on similar topics. The authors are convincing, in my opinion, in their explanation of the need to keep a multilevel model. However, I would suggest further checking analyses with pooled OLS, for instance, while controlling for year and country.

 7. I found tables 4 and 5 quite confusing. From the description of the text, table 4 should refer to attitudes towards both dependent variables (policy and contribution). However, the caption refers just to "contribution". Same with Table 5. I think the description of the text is correct, so the authors should put clear captions and specify in the models which of them refers to one dependent variable and which to the other.

 8. The authors refer to long-term dynamics as "historical" immigration. However, this label sounds a little bit misleading to me, since the variable calculates immigration over the far decade, which includes the 2015 period when the EU experienced a surge in migration flows. I would say that this variable (the average share) rather measures a consolidated, or simply long-term immigration. This way, also the policy impact of the research would gain more value because it would support the expectations of the contact theory concerning the positive effects of migrants' integration. However, on this last point see also comment 9.

 9. The authors correctly point out that even the contact theory "works" better with migrants from the EU. Nonetheless, my interpretation of Figure 2 is that long-term migration from outside the EU has at least non-negative effects on attitudes. I would stress this aspect in the comments, too, if my interpretation is correct.

If applicable, is the statistical analysis and its interpretation appropriate?

Yes

Is the study design appropriate and is the work technically sound?

Yes

Is the work clearly and accurately presented and does it engage with the current literature?

Yes

Are the conclusions drawn adequately supported by the results?

Yes

Are sufficient details of methods and analysis provided to allow replication by others?

Yes

Are all the source data and materials underlying the results available?

Yes

Reviewer Expertise:

Survey research, with a focus on public attitudes towards immigration, solidarity, and sovereignism; Quantitative methods (I apply multilevel modelling, although not very frequently); Territorial inequalities and regional-level differences.

I confirm that I have read this submission and believe that I have an appropriate level of expertise to confirm that it is of an acceptable scientific standard.
